# Performance evaluation of Cerenkov luminescence imaging: a comparison of ^68^Ga with ^18^F

**DOI:** 10.1186/s40658-019-0255-x

**Published:** 2019-10-24

**Authors:** J. olde Heuvel, B. J. de Wit-van der Veen, K. N. Vyas, D. S. Tuch, M. R. Grootendorst, M. P. M. Stokkel, C. H. Slump

**Affiliations:** 1 Department of Nuclear Medicine, Netherlands Cancer Institute, Amsterdam, The Netherlands; 2 Robotics and Mechatronics , Technical Medical Centre, University of Twente, Enschede, The Netherlands; 3 Lightpoint Medical Ltd, Misbourne Works, Waterside, Chesham, HP5 1PE UK

**Keywords:** Cerenkov luminescence imaging, Ga-68 PSMA, Performance evaluation, Intraoperative imaging

## Abstract

**Background:**

Cerenkov Luminescence Imaging (CLI) is an emerging technology for intraoperative margin assessment. Previous research only evaluated radionuclide 18-Fluorine (^18^F); however, for future applications in prostate cancer, 68-Gallium (^68^Ga) seems more suitable, given its higher positron energy. Theoretical calculations predict that ^68^Ga should offer a higher signal-to-noise ratio than ^18^F; this is the first experimental confirmation. The aim of this study is to investigate the technical performance of CLI by comparing ^68^Ga to ^18^F.

**Results:**

The linearity of the system, detection limit, spatial resolution, and uniformity were determined with the LightPath imaging system. All experiments were conducted with clinically relevant activity levels in vitro, using dedicated phantoms. For both radionuclides, a linear relationship between the activity concentration and detected light yield was observed (*R*^2^ = 0.99). ^68^Ga showed approximately 22 times more detectable Cerenkov signal compared to ^18^F. The detectable activity concentration after a 120 s exposure time and 2 × 2 binning of ^18^F was 23.7 kBq/mL and 1.2 kBq/mL for ^68^Ga. The spatial resolution was 1.31 mm for ^18^F and 1.40 mm for ^68^Ga. The coefficient of variance of the uniformity phantom was 0.07 for the central field of view.

**Conclusion:**

^68^Ga was superior over ^18^F in terms of light yield and minimal detection limit. However, as could be expected, the resolution was 0.1 mm less for ^68^Ga. Given the clinical constraints of an acquisition time less than 120 s and a spatial resolution < 2 mm, CLI for intraoperative margin assessment using ^68^Ga could be feasible.

## Background

In cancer surgery, the intraoperative distinction between malignant and healthy tissue is difficult. Incomplete surgical resection heavily impacts patient outcome because of the higher chance of additional therapy and worse prognosis [1–3]. A positive surgical margin (PSM), defined as tumour cells on the inked tissue margin on post-operative histopathology, increases the risk of disease recurrence. In radical prostatectomy, it has been described that 11–38% of patients have a PSM [4–7].

An emerging optical technology in this respect is Cerenkov luminescence imaging (CLI) that visualizes the presence of β-emitting radionuclides by the detection of Cerenkov photons [8–11]. These photons originate when a β particle travels faster than light in a specific dielectric medium [11–13]. The combination of nuclear and optical imaging has great potential for margin assessment as specific radiotracers are developed to target cancerous tissue and the Cerenkov radiation has a superficial penetration depth in tissue. Additionally, CLI has the advantage of using clinically approved imaging agents, which facilitates fast clinical translation of the technology. Academic researchers worldwide have already demonstrated the (pre-)clinical feasibility of CLI for intraoperative specimen analysis [8, 14–16]. The first clinical trial for margin assessment with CLI, performed in breast cancer surgery with ^18^Fluorine-Fluorodeoxyglucose (^18^F-FDG), has shown promising initial results [16].

To date, (pre-)clinical research in CLI has been mostly restricted to the metabolic radiotracer ^18^F-FDG [11]. However, ^18^F-FDG is not suitable as a diagnostic imaging tool for prostate cancer, since prostate cancer is considered hypometabolic and hence, accumulates only limited amounts of FDG [17, 18]. Therefore, the more specific positron emitting radiotracer ^68^Gallium-Prostate-Specific Membrane Antigen (^68^Ga-PSMA) was introduced several years ago. PSMA is a transmembrane protein with significantly elevated expression on prostate cancer cells in comparison with benign prostatic tissue [19, 20]. Furthermore, the isotope ^68^Ga may be advantageous regarding Cerenkov light yield compared to ^18^F because of its higher initial positron energy. This results in more Cerenkov radiation from the positron, due to the energy content itself and the fact that more positrons will reach the Cerenkov threshold. This signal boost can be of importance, due to the generally low signal-to-noise ratio (SNR) of CLI [12]. The higher signal could reduce the injected activity and the acquisition time while reducing the imaging noise. According to Monte-Carlo simulations, ^68^Ga can have theoretically a 26× higher signal yield compared to ^18^F [21]. Nonetheless, it is known that these simulations may not comply with physical experiments [12, 21, 22]. Ciarrocchi et al. suggested as well, after in vitro studies concerning the performance of CLI using ^18^F, to evaluate the impact of different PET radiotracers [23]. Thus, the aim of this research was to evaluate the technical performance of CLI using ^68^Ga and to relate these outcomes to ^18^F. Based on these study outcomes a CLI protocol that will fit clinical needs for margin assessment using ^68^Ga-PSMA during prostate cancer surgery will be proposed.

## Methods

### Experimental design and requirements

The present study was designed to evaluate the performance of CLI comprising two relevant considerations; experiments should be conducted with clinically relevant activity levels and imaging protocols. Next to that, experiments were optimized with the following practical requirements: fast acquisition time, radiation safety, high sensitivity for small lesions, and good tumour to non-tumour distinction. Radioisotopes used in this study were 2-Deoxy-2-(^18^F) Fluoroglucose (IBA Molecular) and ^68^Ga coupled to Glu-urea-Lys (Ahx)-HBED-CC (Scintomics GmbH). The measured activity levels for ^68^Ga-PSMA in prostate cancer were leading for all experiments, and ^18^F activity levels were adapted accordingly.

### Tumour uptake on PET scans

The ^68^Ga-PSMA concentration of 30 primary prostate cancer tumours and benign prostate tissue was measured on a positron emission tomography/computed tomography (PET/CT) in order to conduct the in vitro experiments with clinically relevant radioactivity concentrations. Patients had undergone a positron emission tomography/computed tomography (PET/CT) scan on a Philips Gemini TF system (Philips, Best, the Netherlands), ~ 45 min after an intravenous injection of ~ 100 MBq ^68^Ga-PSMA. Acquisitions were performed from mid-thigh to skull base starting with 3 min per bed position in the pelvic area and 2 min per bed over the remaining body. PET images were reconstructed using BLOB-OS-TF without any post-reconstruction filter, including corrections for decay, random coincidences, dead time, low-dose CT-based attenuation, and scatter. The average tumour uptake in Becquerel (Bq) per millilitre was measured using a spherical VOI placed around the tumour lesion, with a minimal size of 3.3 cm^2^ (Osirix MD DICOM viewer v.9.2, Pixmeo SARL, Bernex, Switzerland).

### Cerenkov luminescence imaging (CLI) system and acquisition settings

The LightPath optical imaging system (Lightpoint Medical Ltd., Chesham, UK) is developed for intraoperative margin assessment using the Cerenkov radiation induced by β-emitting radionuclides. This system is equipped with a camera lens (F/0.95, 512 × 512 pixels) coupled through optics to a − 80 °C cooled electron multiplying charge coupled device (EMCCD; Andor iXon Ultra 897). The EMCCD is shielded with a tungsten plate and folded optics are used to reduce the number of gamma photons striking the EMCCD sensor. A standard optical camera (F/1.4 lens, 1600 × 1200 pixels) was used to acquire white-light reference images. The system has a light-tight imaging chamber to shield from ambient light.

In this study, images were initially acquired using the previously published protocol for ^18^F with an exposure time of 300 s and 8-pixel binning (E300B8) [16]. To find a suitable protocol for clinical application using ^68^Ga, acquisition settings were varied with exposure times of 60, 120, and 300 s without or with the use of 2-, 4-, or 8-pixel binning. Acquisition protocols applied for both radionuclides. Unless otherwise specified, images were acquired without an optical filter. Data analysis was performed using MATLAB R2017b (The MathWorks, Natick, 2017).

### Linearity and detection limit

Three 2-mL Eppendorf tubes of both radionuclides were filled with 2.5, 12.5, and 45 kBq/mL diluted in 1 mL water to investigate the linearity of the system, i.e. to evaluate the correlation between the signal intensity and activity concentration. CLI imaging was performed every 20 min during four subsequent hours and images were processed with the LightPath software, by applying a median filter (3 × 3 pixels) and Gaussian filter (3 pixels) to reduce the noise from high-energy photons, also known as gamma strikes. The latter are 511 keV annihilation photons emitted from the radionuclide, visualized as a local high signal spike with a characteristic tail. After filtering, CLI images were aligned to the white-light reference image.

The radiance (photons/s/cm^2^/sr) per activity concentration was used to compare the signal of both radionuclides. The average radiance was obtained by manually selecting a region of interest (ROI) of 150 pixels around the Cerenkov signal of the Eppendorf tubes, and a ROI outside the Eppendorf tubes to determine the average background signal. A possible linear relationship between radiance and activity concentration will be verified by comparing the radiance half-life (*R*_*t*1/2_) to the decay half-life of both radionuclides. The radiance half-life is the time required to reduce the radiance to half of its initial value.

The radiance per activity concentration was used to determine the detection limit. The lowest detectable activity concentration was defined as the activity concentration where the SNR is 2, which should be sufficient to distinguish the signal in the tumour from the background [24]. The SNR represents difference between the mean signal and the mean background, divided by the standard deviation of the background. The detection limit was determined for both radionuclides and using multiple acquisition settings.

All of the described experiments were repeated under ~ 1-mm raw chicken breast fillet, to simulate the influence of tissue on the signal attenuation and minimal detectable activity concentration. Raw chicken breast was stacked on top of three Eppendorf tubes containing the same activity concentrations as the ones described above.

### Effective spatial resolution

The spatial resolution was determined using glass capillary tubes (outer diameter (OD) 1.1 mm) filled with 33 kBq ^18^F and ^68^Ga. The effect of tissue on the spatial resolution was determined using ~ 1-mm chicken breast fillet on top of the capillaries. The spatial resolution was obtained by averaging intensity profiles perpendicular through cross sections of the imaged line source on raw imaging data without filtering. The average full width half maximum (FWHM) was calculated over 40 mm. Signal intensities were corrected for the zero background level, which was the level of digital counts in the image without radioactivity present. Thereafter, intensities were normalized to correct for possible difference in activity and maximum intensity of ^18^F and ^68^Ga.

### Uniformity

The uniformity of the field of view (FoV) was determined using a square uniform Perspex phantom (60 × 60 mm) with 4.3 MBq ^68^Ga diluted in 15 mL water. Three subsequent images were acquired using an exposure of 60 s without binning. Uniformity was determined on the unfiltered image and after filtering with a median and Gaussian. The median value of three subsequent CLI images was used for analysis, to reduce measurement uncertainty and the effect of the high-energy gamma photons. Uniformity was measured by the mean and standard deviation of the signal by using a ROI over the entire useful field of view (UFoV) and accordingly quantified using the coefficient of variation (CoV). The uniformity of the central field of view (CFoV), defined as 0.75 × UFoV, was determined as well [25].

### Signal-to-background ratio

A signal-to-background ratio (SBR) experiment was conducted to assess the capability of CLI to distinguish tumour from background tissue. The mean ^68^Ga-PSMA concentrations (kBq/mL) of the tumour and the background, derived from the clinical PET scans, were diluted in an Agar solution (0.4 g agar powder in 20 mL of distilled water). Agar is a mixture of agarose and agaropectin, resulting in a gel-like substance. From both solidified Agar solutions, a cube (2 × 2 cm) was excised and exchanged with the other Petri dish (see Fig. 3), and a SBR of 2:1 was obtained. The petri dish was subsequently imaged with different acquisition settings to verify the ability to distinguish the signal. An intensity profile was drawn over the cross section of the Petri dish, to obtain the digital counts per pixel over this line.

## Results

### Tumour uptake on PET scans

The average ^68^Ga-PSMA PET/CT uptake in prostate cancer tumours and in benign tissue is presented in Table 1, showing a large interpatient variation in tumour uptake, tumour volume, and tumour-to-background ratio (TBR). To fit the clinical needs, the CLI should at least detect an activity concentration of 1.59 kBq/mL. In vitro experiments should be conducted with the clinical uptake range of 1.59–8.54 kBq/mL.

**Table 1:** Overview of ^68^Ga-PSMA tumour uptake on PET/CT

	Volume of interest (mL)	Mean tumour uptake (kBq/mL)	Mean uptake tumour × VOI (kBq)	Tumour-to-background ratio
Average	17.4	3.35	80.96	2.7
Standard deviation	11.4	1.53	71.50	1.1
Minimum	3.3	1.59	11.20	1.2
Maximum	53.0	8.45	275.24	5.7
Median	14.3	2.62	51.35	2.5

### Linearity and detection limit

**Fig. 1:**
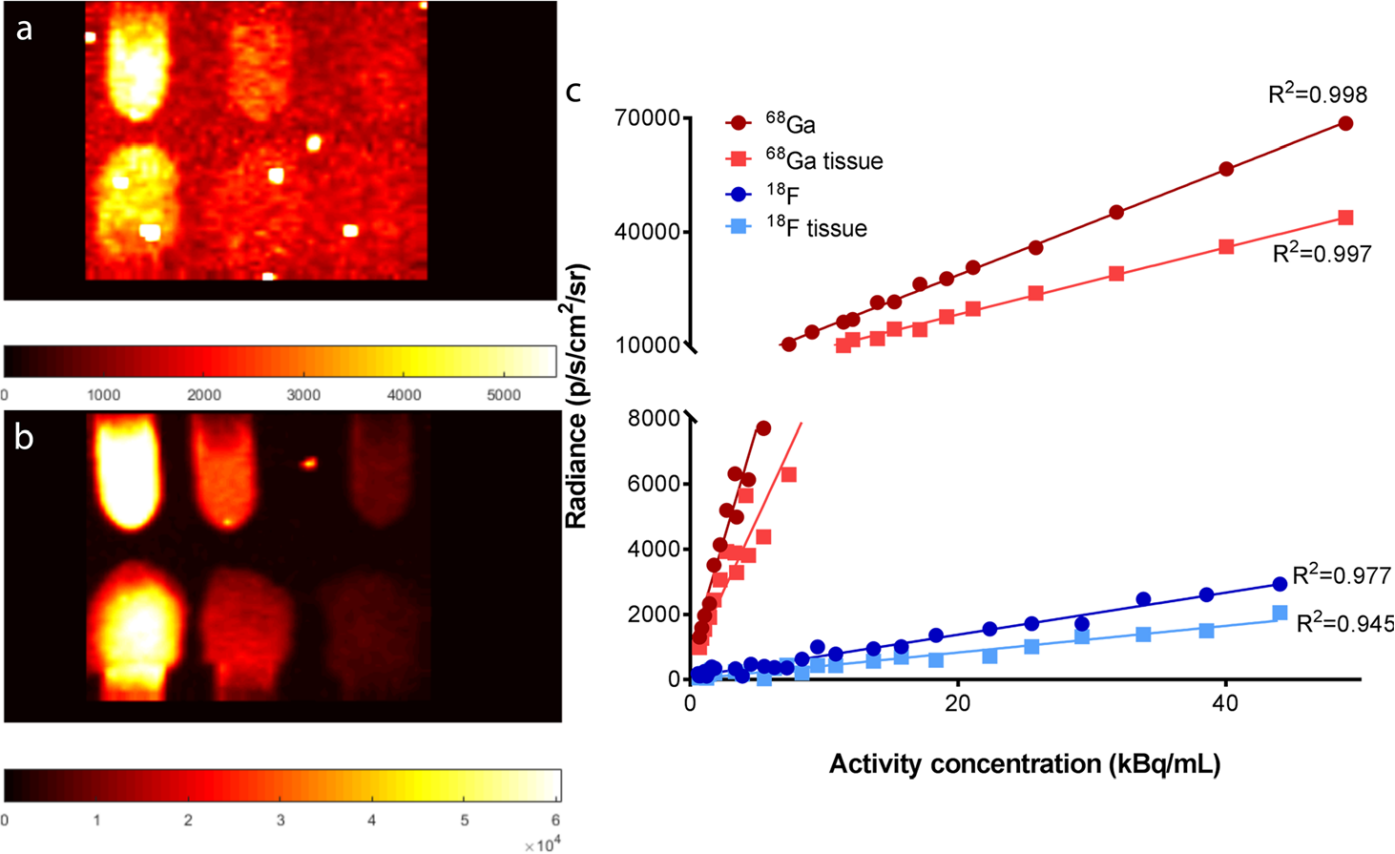
Example of two CLI images of 6 Eppendorf tubes filled with ^18^F (**a**) and ^68^Ga (**b**), where the bottom row is overlayed with 1 mm tissue (**a**). From left to right the tubes are filled with 1 mL of 45, 12.5, and 2.5 kBq/mL activity. (**c**) Effect of tissue on linearity and signal intensity ^18^F and ^68^Ga. Data acquired with an exposure time of 120 s and binning 2 × 2. ^68^Ga gives a 22× higher light yield compared to ^18^F on average. The break line was added to the graph, to visualize the effect of tissue on the ^18^F radiance. Without break line, this was not visible, due to the high radiance of ^68^Ga

For both radionuclides, an excellent linear relationship between the radioactivity concentration and detected light yield (radiance) was observed (^18^F: *R*^2^ = 0.98; ^68^Ga: *R*^2^ = 0.99) for exposure 120 s and binning 2 (E120B2) (Fig. 1). ^68^Ga resulted in approximately 22 times more signal compared to ^18^F with similar imaging settings. CLI signal linearity within the clinical prostate cancer tumour uptake range on ^68^Ga-PSMA PET/CT was for above *R*^2^ = 0.95, whereas ^18^F decreased to *R*^2^ = 0.74 and *R*^2^ = 0.42, without and with tissue surrogate, respectively (Additional file 1: Figure S1). The linear relationship remained with the addition of tissue surrogate on the Eppendorf tubes (^18^F: *R*^2^ = 0.95; ^68^Ga: *R*^2^ = 0.99) (Fig. 1). Although, the signal intensity decreased to 73% and 62% of the original ^18^F and ^68^Ga signal, respectively. Acquisition, with exposure 300 s and binning 8 (E300B8) setting, resulted in a linear relationship of ^18^F: *R*^2^ = 0.97/0.95 and ^68^Ga: *R*^2^ = 0.97/0.93 with and without tissue surrogate, respectively. The radiance half-life of CLI was attained in 115 min for ^18^F (*τ* = 0.006 min^− 1^; *R*^2^ = 0.98) and within 69 min for ^68^Ga (*τ* = 0.01 min^− 1^; *R*^2^ = 0.99), both approximating the radionuclide half-life times of 109 and 68 min, respectively. The detection limit for both radionuclides, with and without tissue surrogate, can be found in Table 2 (see also Additional file 1: Figure S2 for the visual representation of the detection limit).

**Table 2:** Minimal detectable activity concentration (kBq/mL) for ^18^F and ^68^Ga with SNR = 2, using different acquisition protocols

	^18^F		^68^Ga	
Acquisition	300 s and bin 8 × 8	120 s and bin 2 × 2	300 s and bin 8 × 8	120 s and bin 2 × 2
No tissue	3.42	23.66	0.14	1.18
Tissue	5.04	39.16	0.24	1.78

### Effective spatial resolution

**Fig. 2:**
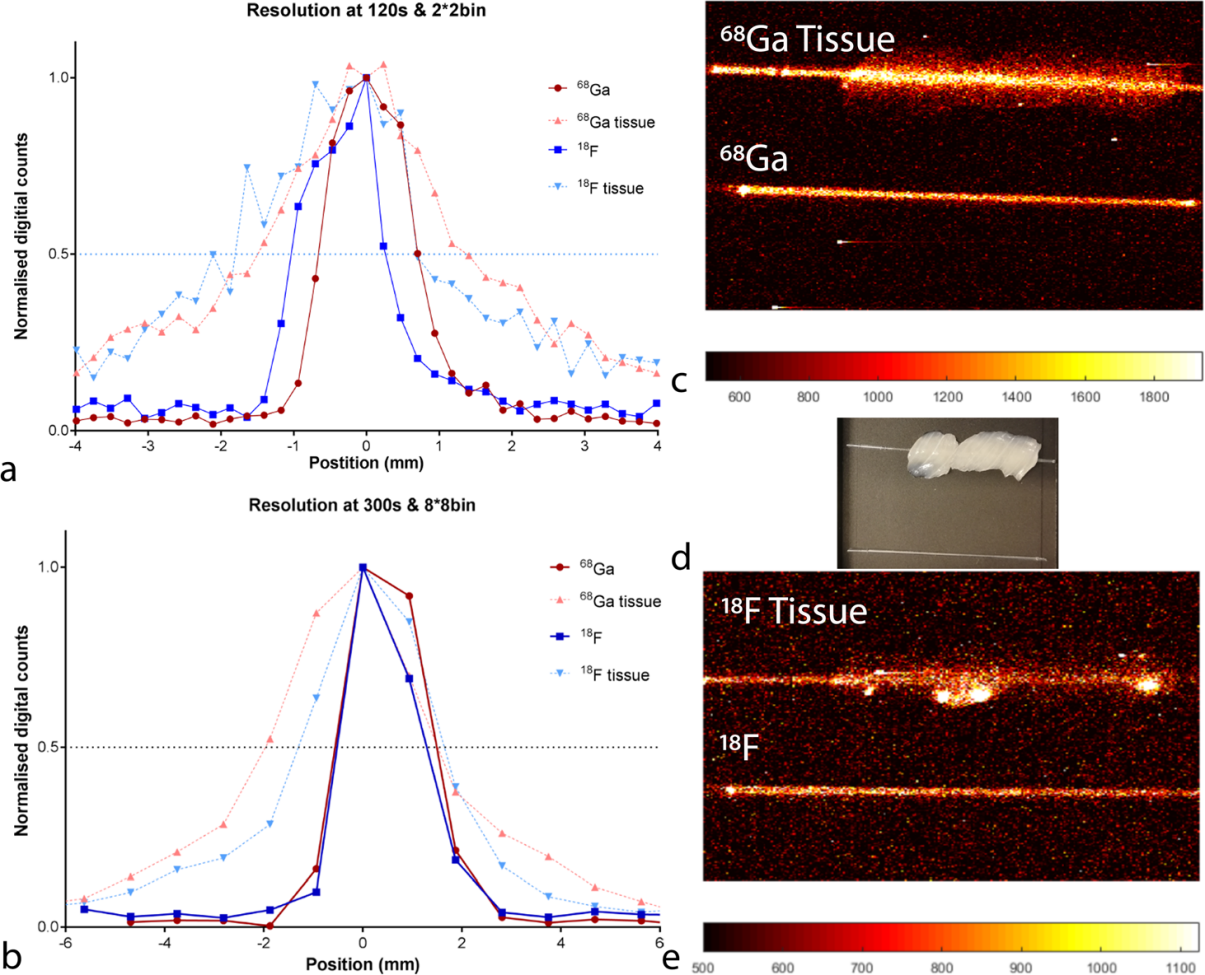
The FWHM of ^18^F (blue) and ^68^Ga (red) for 120 s and 2 × 2 binning (**a**), 300 s and 8 × 8 binning (**b**), the influence of tissue on spatial resolution is displayed in light blue and light red. Signal intensities were normalized, to correct for the difference in activity and intensity of ^18^F and ^68^Ga. (**d**) Shows a white-light image of the glass capillary tubes with and without tissue. In (**c**) the corresponding CLI image of ^68^Ga and in (**e**) the CLI images where the capillaries are filled with ^18^F. The CLI images visualized were used as input to determine the FWHM over a line profile. The displayed images were acquired with 120 s and 2 × 2 binning

The FWHM at E120B2 and E300B8 was for ^18^F 1.31 mm and 1.61, respectively, and for ^68^Ga 1.40 mm and 1.85 mm, ~ 1 mm, respectively (see Fig. 2). The addition of ~ 1-mm tissue surrogate increased the FWHM for both settings 2.40 and 2.85 mm for ^18^F and 2.73 and 3.40 mm for ^68^Ga, respectively.

### Uniformity

The unfiltered median of three uniformity phantom CLI images showed a CoV of 0.18 of both the UFoV and CFoV. After Gaussian and median filtering, the uniformity of the images improved to a CoV of 0.09 in the UFoV and 0.07 in the CFoV (Additional file 1: Figure S3).

### Signal-to-background ratio

**Fig. 3:**
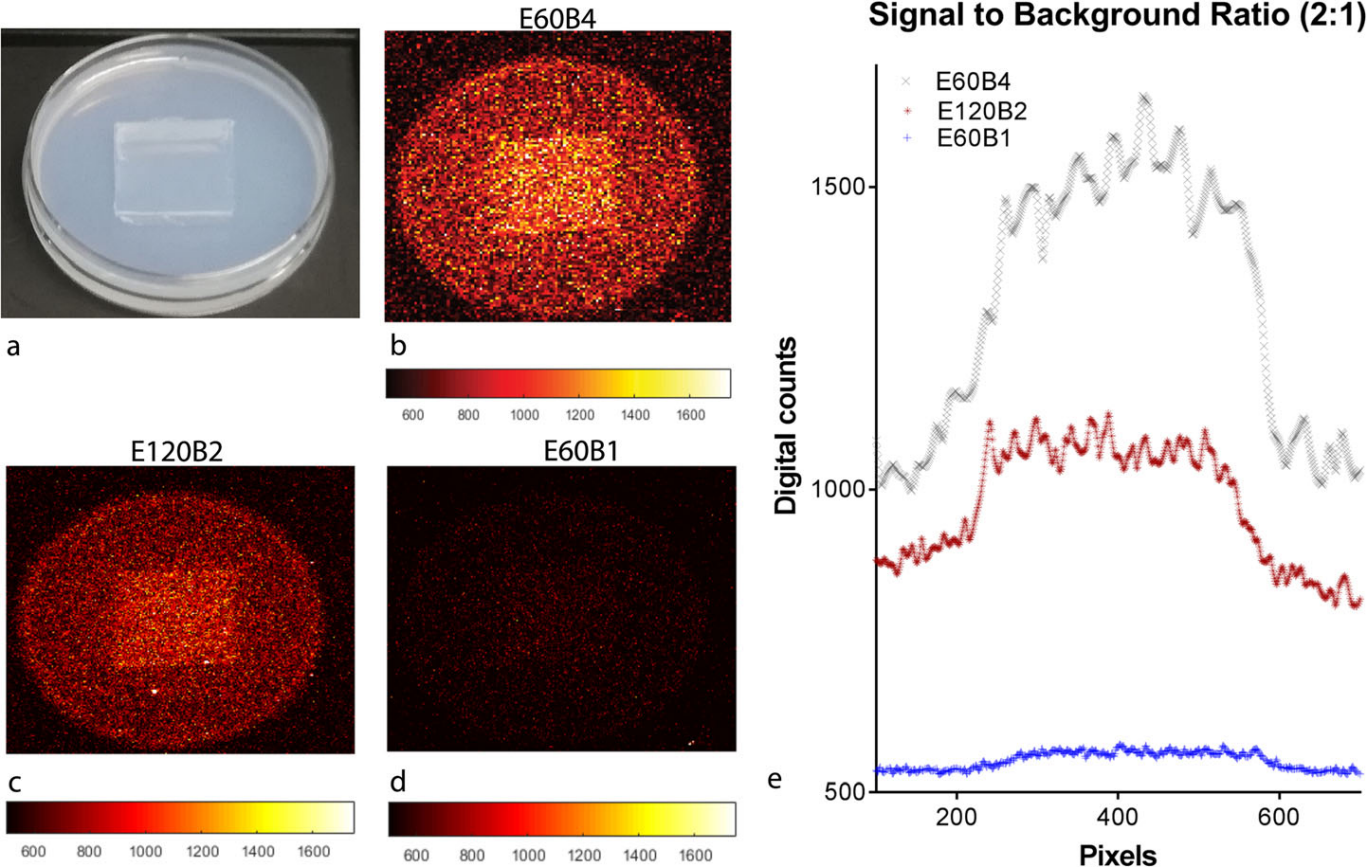
A representative image of the signal-to-background ratio experiment setup (**a**). The result of three different exposure times and binning settings (**b**–**d**). The graph of intensity profile across the previous three Petri dishes (**e**)

Figure 3 shows the results of the signal-to-background experiment, where binning improved the SBR, yet compromised the sharpness of the transition from background to signal. Analysis of the intensity profiles showed a SBR of 2.1 using the E60B4 protocol, which is comparable to the activity concentration ratio of the tumour and background Agar solutions. E60B1 and E120B2 resulted in a SBR of 2.16 and 1.5, respectively.

## Discussion

In this study, the optical properties and performance of CLI using ^68^Ga were compared to those of ^18^F, under clinically relevant activity levels. The radiance was linearly associated with the activity of both radionuclides (^18^F: *R*^2^ = 0.98; ^68^Ga: *R*^2^ = 0.99). The linear relationship persisted with the addition of roughly 1 mm of the tissue surrogate. ^68^Ga showed a 22 times stronger radiance compared to ^18^F in the current study. Glaser et al. simulated the fluence rates with presence of scatter and absorption, to mimic optical properties of tissue, where a higher fluence rate was observed for ^68^Ga [26]. Fan et al. and Beattie et al. compared fluence rates for ^18^F and ^68^Ga measured with IVIS cameras and found radiation rates of 15 and 19 times higher for ^68^Ga [22, 27]. This signal intensity difference between ^18^F and ^68^Ga was slightly lower than the 26 times factor the Monte-Carlo simulations found in the literature [21]. This inconsistency may be explained by certain definitions in Monte-Carlo experiments, such as the use of true point sources in MC-simulations, whereas the relatively larger volume of an Eppendorf tube will radiate from multiple angles onto the camera. Other effects may include machine differences and non-linear detector efficiencies of the different camera systems. Furthermore, in real-life experiments, a fraction of positrons will escape from the medium before emitting Cerenkov photons [12]. Still, our results underline the superiority of ^68^Ga for Cherenkov imaging with respect to signal yield.

Our results indicate that this specific CLI system was not homogeneous over the entire FoV, given the found CoV of 0.18 in the raw image. Visual inspection of the CL image shows a weaker signal near the edges of the uniformity phantom. Though this observation could be due to a commonly encountered phenomenon in optics known as lens vignetting, the results may also have been influenced by the relatively small size of the phantom which matches the 60 × 60 mm FoV exactly. Accordingly, the edges of the phantom are visualized as ‘darker’ due to the lack of positron emissions from the material just outside the FoV. To account for this problem, a uniformity phantom should cover the FoV with an additional margin on all sides that is greater than the positron range. The vignetting in the FoV does not hamper the clinical assessment, since the size of average prostate is small enough to fit in the CFoV [28]. Thus, it is suggested to only use the CFoV to image the specimen. The CoV of the processed image, which is part of the clinical CLI protocol, in the CFoV was 0.07 without binning. This was considered uniform enough to leave out additional post-processing steps to improve the uniformity. The experiment was only performed with ^68^Ga, since uniformity of the system is expected to be independent of the radionuclide. However, the larger positron range could alter the texture of the image in comparison to a shorter range.

The resolution response was determined using a glass capillary with an outer diameter of 1.1 mm. Though the camera is able to image up to 158 μm according to the specifications, a better resolution is not deemed clinically relevant, as the surgeon is not able to resect with a higher accuracy. Still, we believe that difference in resolution between ^18^F and ^68^Ga found in this study is not entirely trivial, especially when the tissue surrogate was added. At higher binning (E300B8) this difference with tissue was roughly 0.5 mm. If a PSM occurs, surgeons are only able to shave in a specific area surrounding the PSM. Though this shaving cannot be performed with submillimetre precision, the localisation of the suspected area should be as precise as possible. That being said, a PSM found on ex vivo measurement is difficult to map back in vivo; therefore, the lower resolution of ^68^Ga will not hamper clinical implementation. The smaller resolution of ^18^F found in this study complies with literature [12, 22, 29] and can be explained by the larger positron range of ^68^Ga compared to ^18^F [30]. Direct comparison of the spatial resolution is difficult, since other groups used different setups to determine the effective spatial resolution [22, 23]. Binning is an important factor that influences the spatial resolution, since data of different pixels is combined to enhance the signal and reduce the effects of noise. Although a binning of 2 reduces the spatial resolution with roughly 10% (1.08–1.16 mm, for a 60-s exposure), the gain in signal intensity is fourfold, thus justifying the use of pixel binning in a clinical setting.

To come to a clinical image acquisition protocol that could be used during prostatectomy, various acquisition times and pixel binning setting were evaluated. The most optimal setting for clinical ex vivo ^68^Ga research is considered 120 s and 2 × 2 binning, thus acquiring a good spatial resolution within an acceptable timeframe for intraoperative usage and sufficient sensitivity. This is a shorter exposure time and binning factor, as the default setting for ^18^F (E300B8) [16, 23]. Since the light yield is higher with ^68^Ga, the acquisition time and binning factor can be decreased without compromising the quality of the image. For clinical implementation, time is important. For rapid assessment, the operation cannot be delayed for more than 10 min. Uptake in the prostate tumour, from analysis, shows that the uptake is sufficient for CLI imaging with ^68^Ga-PSMA, with the required time for prostatectomy and lesion removal.

### Implications for CLI during prostatectomy

^68^Ga-PSMA tumour uptake measurements were performed in a heterogeneous group of prostate cancer patients. Although large variations are observed in the intensity of PSMA accumulation, we have determined an average and minimal uptake to enable clinically relevant measurements. It was stated that CLI should be able to visualize an average concentration of 3.35 kBq/mL. Based on our in vitro results the detection limit and contrast for ^68^Ga (with and without tissue) is sufficient to detect this average tumour uptake, even with an exposure time as low as 120 s. The detectable activity concentration with 1-mm tissue asks for an injection of 2.6 MBq/kg ^68^Ga 45 min prior to CLI, assuming a uniform distribution and water density in the body. Nevertheless, the tumour has 100× more receptors as benign tissue [19, 20]; thus, injected dose could be lowered for CLI visualization, thereby complying with the ^68^Ga-PSMA guidelines for PET imaging [31]. The standard clinical injection of ~ 100 MBq would be sufficient for intraoperative application with a protocol that fits the clinical requirements. When increasing either the binning or the exposure time, potentially an even lower radioactive dosage can be used. Still, precise patient dosage will be determined in our on-going clinical feasibility study. Prior studies with ^18^F showed that the radiation dose to the surgeon due to the CLI procedure was 34 μSv per procedure and 2–20 μSv per scrub nurse [14, 16]. The use of ^68^Ga-PSMA decreases the injected dose and thus improves the radiation safety for both the patient and personnel.

The Cerenkov signal through ~ 1-mm tissue surrogate reduced to 73% of the original signal for ^18^F, and 62% for ^68^Ga according to our measurements. However, this experimental setting does not mimic the exact clinical situation as the chicken breast was not perfused and the optical properties do not comply. The signal is influenced by scattering and absorption in tissue, the attenuation found in the current study (73%), approximates the value found in literature. Theoretical calculations of 1-mm tissue, resulted in a decrease in signal intensity of ^18^F of 77% [32]. The same approximation was also made for higher energy nuclides like ^68^Ga. However, the addition of 1-mm surrogate tissue resulted in more signal decay in our experiments (62%) than expected from literature (77%). Difference in this attenuation percentage could be explained by the influence of the refractive index (η). Tissue has a higher η resulting in a higher number of Cerenkov photons produced (see Eq. 1). The corresponding higher mass density results in a higher β attenuation cross section and concomitant reduced β particle range. Increased density therefore tends to reduce Cerenkov radiation production efficiency, but for radionuclides that emit relatively low energy β’s, the increased η dominates resulting in higher light yield for higher density materials. For the high-energy β’s of ^68^Ga, however, the impact of η is small and the reduction in light yield due to the density effect dominates [22]. Additionally, Glaser et al. showed that a larger refractive index has more impact on the fluence rate of ^18^F, as ^68^Ga [26].

Equation 1 is the number of Cerenkov photons *N* emitted per distance travelled *x*, which is derived from the Frank–Tamm equation [21]. *β* = particle velocity, η = refractive index, λ = wavelength (nm), and α = fine structure constant (*α* ≈ 1/137).

$$ \frac{\mathrm{d}N}{\mathrm{d}x}=2\pi \alpha \left(1-\frac{1}{\beta^2{\eta}^2}\right)\left(\frac{1}{\lambda_1}-\frac{1}{\lambda_2}\right) $$

### Limitations of CLI

The interference of tissue and blood reduces the obtained signal; however, these influences were not simulated in the current in vitro set up. Therefore, it is difficult to suggest a definite ex vivo acquisition protocol upon solely in vitro measurements. Cerenkov light is predominant in the blue range of the spectrum, and attenuates towards the red part of the spectrum [33]. The weight of the spectrum changes with the influence of tissue, since the blue part is more strongly absorbed [10] as haemoglobin absorbs mostly in this part of the spectrum. For margin assessment accuracy in the penetration, depth is important, although not considered in this study. To estimate the penetration depth, it is important to have a phantom with the optical properties of the prostate, since it is influenced by scattering and absorption. The development of a prostate-like-phantom was outside the scope of this paper. For clinical application, the use of numerous filters is recommended, since it enables the possibility to only obtain emitted photons generated near the surface of the tissue. Filters would be needed to determine the depth of the lesion, as the tissue scatters. Scattering is more present in tissue and has a larger influence on light from longer distances [34]. The resulting attenuation is considered positive for margin assessment using CLI, as it benefits superficial imaging to guide complete tumour resection.

## Conclusion

The performance of the CLI system was determined in vitro using both ^68^Ga and ^18^F. The system is linear for both radionuclides at clinically relevant radioactivity concentrations, considering uptake in prostate tumours. ^68^Ga was superior over ^18^F in terms of light yield and detectable activity concentration, with and without the addition of chicken tissue. However, as could be expected, the spatial resolution was lower for ^68^Ga. Still, in combination with the short acquisition time (120 s) and clinically sufficient detection limit (1.8 kBq/mL), it seems feasible to obtain ex vivo CLI images for intraoperative margin assessment during prostate cancer surgery using ^68^Ga-PSMA. Future studies should confirm the feasibility of this system as intraoperative margin assessor by comparison with histopathology.

## Supplementary information

Additional file 1: **Figure S1.** Effect of tissue on linearity and signal intensity ^18^F and ^68^Ga within the range of ^68^Ga-PSMA uptake of the prostate tumour according to measurement on the PET/CT scans. Data was acquired with an exposure time of 120 s and binning 2 × 2. The goodness-of-fit (R^2^) is displayed at every fit. **Figure S2.** Graph representing the signal and the noise floor of ^18^F (top) and ^68^Ga (bottom), with and without overlying tissue. The crossing of the signal with background correction (μs-μb) and standard deviation of the background signal (σb) represents the minimal detectable activity concentration for SNR = 1. CPS = counts per second. **Figure S3.** Three subsequent images of the uniform flood source (A-C) and the image reconstructed of the median values of the three raw images (D), and the three post-processed images (E-G), with the reconstructed median image (H). The use of the median value (H) reduced the influence of gamma strikes (yellow stripes at the red arrows) in E-F. The same intensity scaling was used in all eight images. (PDF 731 kb)

## Data Availability

The datasets used and/or analysed during the current study are available from the corresponding author on reasonable request.

## Abbreviations

^18^F: 18-Fluorine
^18^F-FDG: 18-Fluorine-Fluorodeoxyglucose
^68^Ga: 68-Gallium
^68^Ga-PSMA: 68-Gallium-Prostate-Specific Membrane Antigen
CFoV: Central field of view
CLI: Cerenkov luminescence imaging
CoV: Coefficient of variation
E120B2: exposure time 120 s and binning 2
E300B8: Exposure time 300 s and binning 8
EMCCD: Electron multiplying charge coupled device
FoV: Field of view
FWHM: Full width half maximum
OD: Outer diameter
PSM: Positive surgical margin
ROI: Region of interest
R_*t*1/2_: Radiance half-life
SBR: Signal-to-background ratio
SNR: Signal-to-noise ratio
TBR: Tumour-to-background ratio
UFoV: Useful field of view
