# Supplementary material for: Performance evaluation of Cerenkov luminescence imaging: a comparison of 68Ga with 18F
Source: EJNMMI Phys. 2019 Oct 24;6:17. doi: 10.1186/s40658-019-0255-x (PMC6813407; doi:10.1186/s40658-019-0255-x)
Supplement: Supplementary file 1 — Figure S1. Effect of tissue on linearity and signal intensity 18F and 68Ga within the range of 68Ga-PSMA uptake of the prostate tumour according to measurement on the PET/CT scans. Data was acquired with an exposure time of 120 s and binning 2 × 2. The goodness-of-fit (R2) is displayed at every fit. Figure S2. Graph representing the signal and the noise floor of 18F (top) and 68Ga (bottom), with and without overlying tissue. The crossing of the signal with background correction (μs-μb) and standard deviation of the background signal (σb) represents the minimal detectable activity concentration for SNR = 1. CPS = counts per second. Figure S3. Three subsequent images of the uniform flood source (A-C) and the image reconstructed of the median values of the three raw images (D), and the three post-processed images (E-G), with the reconstructed median image (H). The use of the median value (H) reduced the influence of gamma strikes (yellow stripes at the red arrows) in E-F. The same intensity scaling was used in all eight images. (PDF 731 kb) [file 40658_2019_255_MOESM1_ESM.pdf]

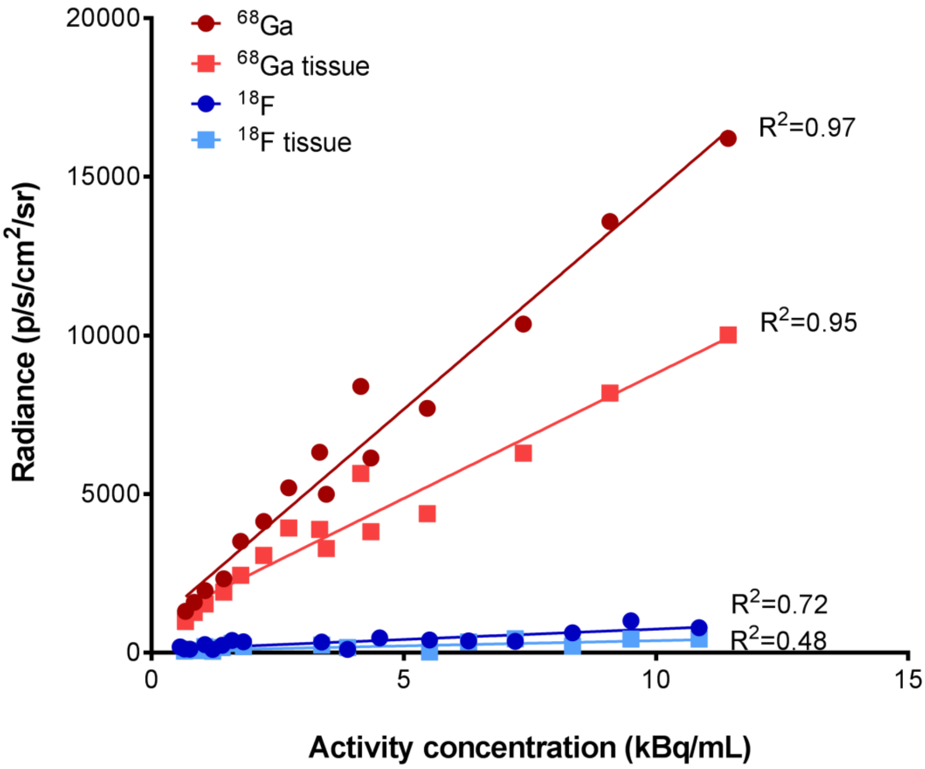

Figure S1: Effect of tissue on linearity and signal intensity ^{18}F and ^{68}Ga within the range of ^{68}Ga-PSMA uptake of the prostate tumour according to measurement on the PET/CT scans. Data was acquired with an exposure time of 120s and binning 2×2. The goodness-of-fit (R^2) is displayed at every fit.

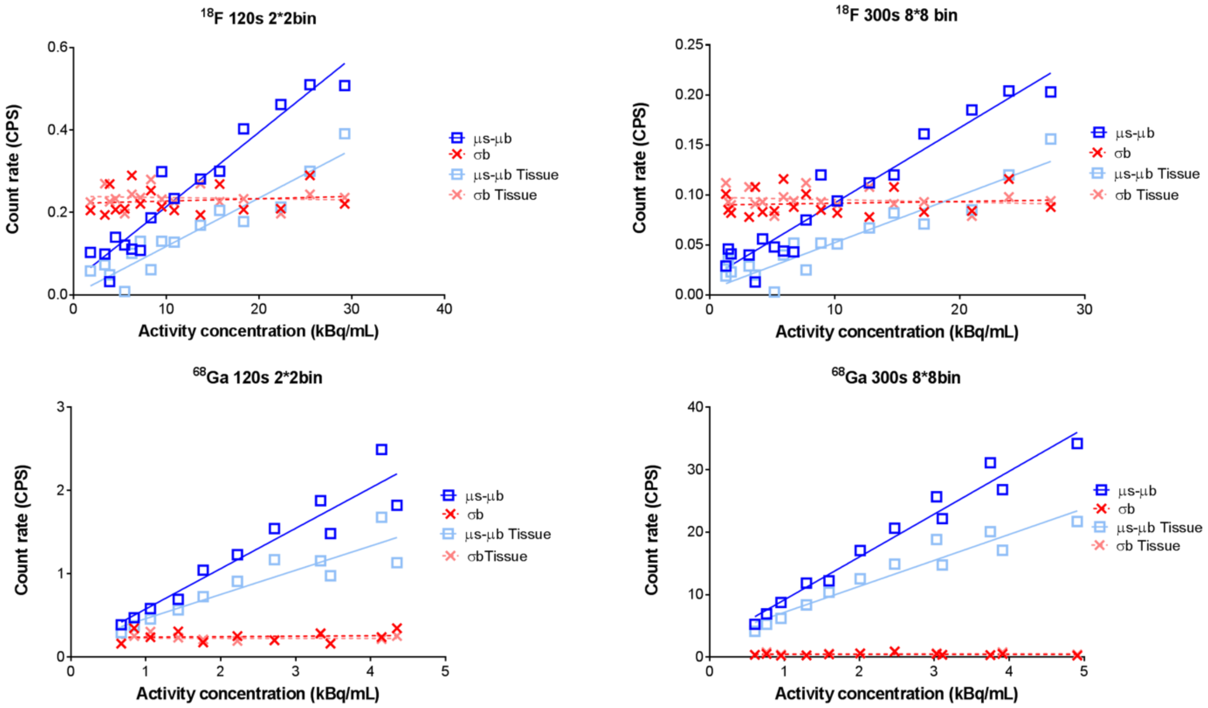

Figure S2: Graph representing the signal and the noise floor of ^{18}F (top) and ^{68}Ga (bottom), with and without overlying tissue. The crossing of the signal with background correction (µs-µb) and standard deviation of the background signal (σb) represents the minimal detectable activity concentration for SNR=1. CPS= counts per second

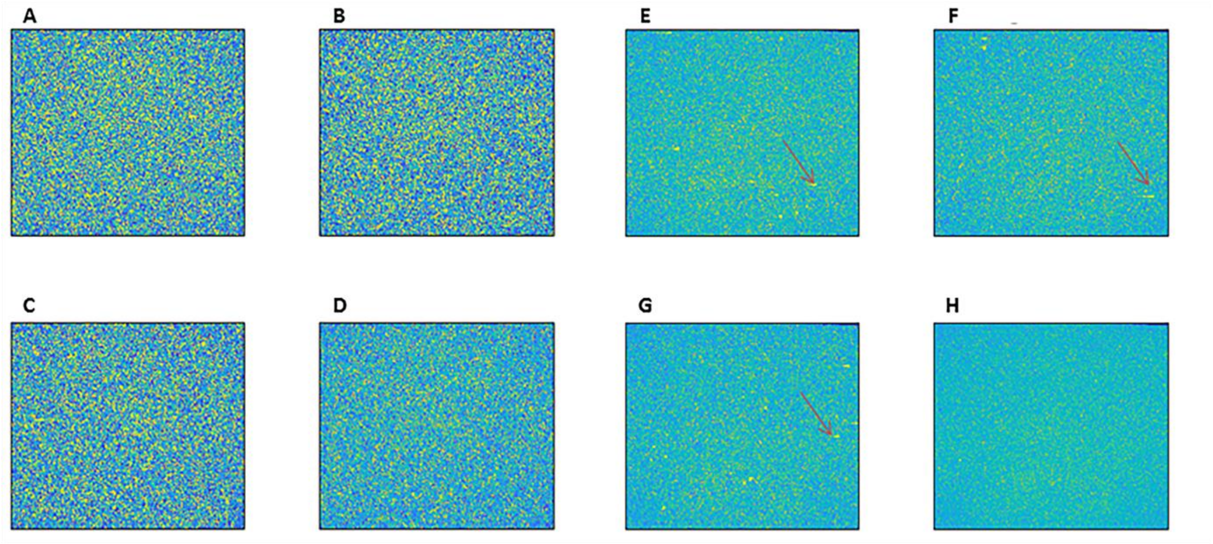

Figure S3: Three subsequent images of the uniform flood source (A-C) and the image reconstructed of the median values of the three raw images (D), and the three post-processed images (E-G), with the reconstructed median image (H). The use of the median value (H) reduced the influence of gamma strikes (yellow stripes at the red arrows) in E-F. The same intensity scaling was used in all eight images.

Title

Performance evaluation of Cerenkov Luminescence Imaging: A comparison of ^{68}Ga with ^{18}F

Journal Name

European Journal of Nuclear Medicine and Molecular Imaging Physics

Authors

J. olde Heuvel[1,2], B.J. de Wit-van der Veen[1], K.N. Vyas[3], D.S. Tuch[3], M.R. Grootendorst[3], M.P.M. Stokkel[1], C.H. Slump[2]

[1] Department of Nuclear Medicine, The Netherlands Cancer Institute-Antoni van Leeuwenhoek, Amsterdam, The Netherlands.

[2] MIRA Institute for Biomedical Technology and Technical Medicine, University of Twente, Enschede, The Netherlands

[3] Lightpoint Medical Ltd, Misbourne Works, Waterside, Chesham HP5 1PE, United Kingdom

Corresponding author:

J. olde Heuvel – j.olde.heuvel@nki.nl
